# Alcohol Consumption during Adolescence and Risk of Diabetes in Young Adulthood

**DOI:** 10.1155/2014/795741

**Published:** 2014-03-17

**Authors:** Wenbin Liang, Tanya Chikritzhs

**Affiliations:** National Drug Research Institute, Curtin University, GPO Box U1987, Perth, WA 6845, Australia

## Abstract

*Background*. There is very limited data available on the association between underage drinking and risk of diabetes. The aim of this study is to investigate the association between alcohol use during adolescence and the risk of diabetes while controlling for a wide range of confounders, including parental alcohol use. *Methods*. This population-based study used data collected from the National Longitudinal Study of Adolescent Health (Add Health). Participants were initially recruited in 1994-1995 (Wave I), then followed up in 1996 (Wave II) and in 2001-2002 (Wave III), and in 2008-2009 (Wave IV). Analysis included 2,850 participants (46% male) who were successfully followed up at Waves I, III, and IV without a known diagnosis of diabetes at Waves I and III and who provided all necessary information for the analysis. *Results*. During adolescence, frequent alcohol consumption at levels reaching 5 or more drinks, 3–7 days/week, substantially increased the risk of diabetes in young adulthood, with an odds ratio of 12.57 (95% CI 4.10–38.61) compared to current abstainers. *Conclusions*. Heavy alcohol use during adolescence may increase the risk of diabetes in young adulthood. *The Significant finding of the Study*.

## 1. Introduction

Diabetes mellitus has become a major global public health problem [1, 2]. It has been estimated that diabetes currently affects 347 million people worldwide [2]. As a chronic condition, diabetes can lead to serious complications such as cardiovascular disease, nephropathy, and infection which are major direct causes of morbidity and mortality [1]. Type 2 diabetes accounts for more than 90% of diabetes cases [2]. Overweight, obesity, and being physically inactive are major risk factors for type 2 diabetes; however, prevention can be achieved by lifestyle modification such as eating a balanced diet and taking regular physical activity [3, 4].

Alcohol use, a common social behavior in most parts of the world, has been investigated for its possible role in relation to the onset of diabetes in a number of observational studies [5, 6]. A relatively recent meta-analysis of cohort studies which have investigated the association between alcohol consumption and diabetes risk showed that, compared to lifetime abstainers, moderate drinkers had a reduced risk of diabetes [7]. However, this observed protective effect has been demonstrated to be moderated, at least in part, by the residual confounding effects of socioeconomic, behavioural, psychological, and genetic factors [7–10]. Nearly all published studies only included participants who were at least 30 years of age at baseline [7]. As childhood obesity and underage drinking have become increasingly common over the last few decades [11, 12], it is important to investigate whether alcohol use among adolescents may affect the risk of diabetes. The aim of this study is to investigate the association between alcohol use during adolescence and the risk of diabetes while controlling for a wide range of confounders, including parental alcohol use.

## 2. Method

This study used data collected from the National Longitudinal Study of Adolescent Health (Add Health). Details of the Add Health study have been described previously [13] and can be accessed at the Add Health website: http://www.cpc.unc.edu/projects/addhealth. Briefly, Add Health is a longitudinal study with a representative sample of adolescents in grades 7–12 (grades equivalent to the Australian education system) at baseline. Participants were initially recruited in 1994-1995 (Wave I, mean age 15.75) and then followed up in 1996 (Wave II, mean age 16), in 2001- 2002 (Wave III, mean age 22.16), and in 2007-2008 (Wave IV, mean age 28.67). A combination of self-administered questionnaires and interviews was employed to collect social, psychological, and health information, including demographics, risk behaviors, health status, and family composition. In addition, the Wave I survey also included a face-to-face interview with one parent (preferably the mother) of each participant to provide further information on the adolescent and other family members, including health problems and lifestyles. In addition to interview, the Wave IV survey collected biological measures of glucose homeostasis including glucose and hemoglobin A1c (HbA1c, %) [14]. The current study used public-use data collected from Wave I, Wave III, and Wave IV of the Add Health study. Only participants who were successfully followed up in Waves III and IV and who provided information for all variables required in the analysis were included in the study (i.e., there were no missing values).

Alcohol consumption during adolescence and young adulthood was determined based on the survey data collected in Waves I and III. In Wave I, participants were asked, “Have you had a drink of beer, wine, or liquor—not just a sip or a taste of someone else's drink—more than 2 or 3 times in your life.” In Wave III, participants were asked the same question about their drinking since Wave I (June, 1995). If, in either wave, the answer was yes, the participant was then asked, “During the past 12 months, on how many days did you drink alcohol?” If the answer was more than one or two day(s), they were further asked about how many drinks they usually consumed each time they drank; that is, “Think of all the times you have had a drink during the past 12 months. How many drinks did you usually have each time? (A “drink” is a glass of wine, a can of beer, a wine cooler, a shot glass of liquor, or a mixed drink.)” Based on answers to these questions, we coded participants according to the frequency and quantity of their alcohol use. Frequency was recoded into five levels based on the original eight categories applied in the survey: (i) lifetime abstainers who never had 2 drinks or more over their lifetime, (ii) those who had 2 drinks or more over their lifetime but not in the last 12 months, (iii) those who drank 1–12 days/year, (iv) those who drank 2–8 days/month, and (v) those who drank 3–7 days/week. Quantity of alcohol use per occasion was measured by number of drinks in the original questionnaire. The Centers for Disease Control and Prevention defined heavy alcohol use or binge drinking as 5 drinks or more per occasion (http://www.cdc.gov). Therefore, in this study, the average quantity of alcohol drunk per occasion was defined as follows: (i) none; (ii) 1–4 drinks; and (iii) 5+ drinks. The same definitions were used for alcohol consumption at Waves I and III.

Diabetic status was determined in Wave IV; participants who reported having diabetes at Wave I and/or Wave III were excluded from the analysis. This study used the same definition of diabetes as the Add Health study: a participant was considered to have diabetes if they had a fasting glucose ≥126 mg/dL (7 mmol/L), or nonfasting glucose ≥200 mg/dL (11 mmol/L), or HbA1c ≥6.5%, or self-reported history of diabetes (except during pregnancy) or used antidiabetic medication in the past four weeks [14].

### 2.1. Data Analysis

We created a variable each for Wave I and Wave III which represented the matrix of drinking frequency by quantity among participants at each survey wave: (i) 1–12 days/year, 5+ drinks/occasion; (ii) 1–12 days/year, <5 drinks/occasion; (iii) 2–8 days/month, 5+ drinks/occasion; (iv) 2–8 days/month, <5 drinks/occasion; (v) 3–7 days/week, <5 drinks/occasion; and (vi) 3–7 days/week, 5+ drinks/occasion.

Multivariate logistic regression was used to investigate the association between alcohol use during adolescence and risk of diabetes in young adulthood. Information on a number of potential confounding factors was obtained from the surveys and controlled for in multivariate analyses, including gender, age at Wave IV, race, BMI (calculated from measured weight and height) at Wave III, parental self-reported diabetic status, frequency of parental alcohol use (from Wave I), smoking status at Waves I and III, urbanity, and median income of residential location at Wave I (variables controlled for are shown in Table A1; see Supplementary Material available online at http://dx.doi.org/10.1155/2014/795741). In order to further control for residual confounding effects, a proxy outcome (parental self-reported diabetic status) was used to estimate the effects of residual confounding effects. Similar approach has been used to examine the “protective” effect of moderate alcohol use [8, 15]. This was achieved by using parental self-reported diabetic status as the outcome variable instead of diabetic status of the index participant, while keeping all the controlled variables unchanged but excluding parental self-reported diabetic status from the controlled variable list. This approach assumes that alcohol use by participants has no physiological effects on their parental diabetic status, while a person's diabetes status and his/her parental diabetic status share a similar set of confounding factors. Any association between parental diabetic status and alcohol use was in fact manipulated by residual confounding effects. If significant residual confounding effects were observed, then the observed residual effects will be offset in the final model. STATA Statistical Software: Release 11 developed by StataCorp LP was used to perform all analyses. Sampling weight provided by the Add Health study was assigned as “the sampling weight” in multivariate logistic regression as indicated in the guideline for analysis of the Add Health Data [16].

## 3. Results

Analyses included 2,850 participants who were successfully followed up at Waves I, III, and IV without a known diagnosis of diabetes at Waves I and III and who provided valid information on all measures (i.e., including alcohol consumption and potential confounders). This constituted 67% of the participants who were successfully followed up at Waves I, III, and IV. The mean (median) age at Waves I, III, and IV was 15.75 (15.79), 22.16 (22.15), and 28.67 (28.66), respectively. 6.0% of these participants had developed diabetes by Wave IV (Table 1). No significant risk difference was observed between males and females. When compared to white Americans, African Americans had significantly higher risk of diabetes (Table A1).

In the first multivariate analysis, adolescents who reported never having had any alcohol (lifetime abstainers) were used as the reference group (Table A1). The analyses were then repeated using adolescents who reported ever consuming alcohol but none in the past 12 months as the reference group, that is, current abstainers (Table 2). As shown in Table A1, at Wave I, both (i) current drinkers who consumed alcohol 2–8 days/month and 5+ drinks on average per occasion (OR = 0.20, incidence rate = 4.55 per 1,000 person-years) and (ii) current abstainers (OR = 0.34, incidence rate = 6.49 per 1,000 person-years) had lower risks of diabetes compared to lifetime abstainers (incidence rate = 11.69 per 1,000 person-years). However, adolescents who consumed alcohol 3–7 days/week and an average of 5+ drinks per occasion had a significantly higher risk of diabetes compared to lifetime abstainers (OR = 4.24, incidence rate = 38.01 per 1,000 person-years). No significant association was observed between alcohol consumption and risk of diabetes at Wave III when lifetime abstainers were the reference group (Table A1).

It was also apparent that adolescents at Wave I whose fathers had not consumed alcohol in the last 12 months had significantly higher risk of having developed diabetes by Wave IV compared to those whose fathers consumed alcohol once or less per month (OR = 2.29). Adolescents whose mothers consumed alcohol 2 to 3 times per month had significantly lower risk of diabetes compared to those whose mothers consumed alcohol once or less frequently per month (OR = 0.34) (Table A1).

When current abstainers were chosen as the reference group (no alcohol in the past 12 months), no significant inverse association (i.e., protective effect) was found for alcohol use and risk of diabetes. Instead, adolescents (i) who drank more than three days per week and 5+ drinks per occasion on average (OR = 12.57) and (ii) those who drank alcohol 1–12 days per year and less than 5 drinks per occasion (OR = 2.46) had significantly higher risks of diabetes in young adulthood. At Wave III, when participants aged a little over 22 years on average, there was no indication of any significant association between alcohol consumption and risk of diabetes (Table 2).

The association between alcohol use of index participants and proxy outcome (parental self-reported diabetic status) was only significant for current abstainers comparing to lifetime abstainers at Wave III, while no residual confounding effect was detected for other categories (Table A2). When the residual confounding effect (natural logarithm of 2.56) was offset for current abstainers at Wave III, the odds ratio (95% confidence interval) became 0.25 (0.09, 0.66). This estimate is similar to the odds ratio of current abstainers at Wave I (Table A1).

## 4. Discussion

To the best of our knowledge, this is the first study to have specifically investigated the effect of alcohol use in adolescence on the risk of diabetes in young adulthood (i.e., under 30 yrs). Frequent heavy alcohol use at an early age (i.e., under the age of 16 yrs and consuming an average of 5+ drinks on 3 or more days/week) was associated with significantly increased risk of diabetes: OR = 4.24, incidence rate increased to 38.01 per 1,000 person-years when compared to lifetime abstainers (incidence rate = 11.69 per 1,000 person-years), and OR = 12.57 when compared to current abstainers (incidence rate = 6.49 per 1,000 person-years). It is well established that excessive alcohol use can lead to various forms of liver disease [17, 18] as well as chronic or acute pancreatitis [19, 20]. Both the liver and the pancreas play major roles in controlling plasma glucose levels [21]. It is plausible that the strong association between frequent risky drinking and the onset of diabetes observed in this study is at least partly mediated by liver and pancreatic injuries induced by alcohol at a time of rapid physiological growth and development.

It was observed that, for all frequency categories, consuming less than 5 drinks per occasion at Wave I was not significantly associated with reduced risk of diabetes. It was also observed that adolescents who consumed alcohol 2–8 days per month and 5+ drinks per occasion were significantly associated with lower risk of diabetes compared to those who had never consumed alcohol in their lifetime, but there was no significant difference when compared to those who had not consumed alcohol in the past 12 months (i.e., non-lifetime, current abstainers). Furthermore, it was observed that current abstainers at Wave I have lower risk of diabetes comparing to lifetime abstainers. It is impossible to rule out the possibility that infrequent high level alcohol use may produce biological protective effect against diabetes in adolescents; however, these findings indicated that the observed protective effect for infrequent alcohol consumption at high level (5+ drinks per occasion) is more likely to be mediated by genetic and social factors that strongly associate with alcohol use and health [10].

The likely presence of residual confounding effects was also indicated by significant associations between parental alcohol consumption and risk of diabetes among the participants, which cannot be explained by the physiological effects of alcohol. Adolescents who were able to tolerate more than 5+ drinks per occasion while maintaining an infrequent consumption pattern may be genetically different from lifetime abstainers. It has been suspected for many years that genetic predisposition may be involved in the association between moderate alcohol consumption and risk of diabetes demonstrated by observational studies [22]. For instance, variations in genes encoding alcohol dehydrogenase (ADH) and aldehyde dehydrogenase (ALDH) (enzymes which are responsible for ethanol metabolism) have been found to play an important role in determining drinking patterns [23]. After absorption, ethanol is oxidized to acetaldehyde by ADH and acetaldehyde is then converted into acetate by ALDH, and, therefore, for a given amount of alcohol intake, the concentration of acetaldehyde in different people is largely determined by the variants of ADH and ALDH [24–26]. Acetaldehyde is responsible for many of the unpleasant side effects of alcohol use such as flushing, nausea, and dizziness, which in turn can deter people from drinking [24, 25, 27]. Recent research has provided some evidence to suggest that ADH and ALDH variants may modify the association between alcohol consumption and diabetes [28–30] and coronary heart disease [31]. For example, Beulens et al. showed that the “protective effects” of alcohol consumption on diabetes were reduced among participants carrying ADH alleles related to slower oxidation of ethanol [28]. It is possible that inverse associations which appear between adolescent risky drinking and the risk of diabetes in adulthood are due to genetic predisposition, rather than the protective effects of alcohol use itself. For instance, a recent study by Ng Fat and Shelton showed that young adults who had chronic illness were less likely to consume alcohol [32]. The findings of the current study also concur with recently published analyses of the National Health Interview Surveys (USA) showing inverse associations between light and moderate alcohol consumption of key participants and the prevalence of adverse health status among their family members, including children, which is indicative of residual confounding [33].

There are some limitations to the current study. We were unable to distinguish between type 1 and type 2 diabetes. About 30% of participants were excluded from the analysis due to missing information on one or more variables. Level of physical activities and dietary intake was not controlled in this study. This study was also subject to recall bias (tendency toward underestimation) which is common to almost all published cohort studies on alcohol and chronic disease which rely on participant self-reported alcohol use. To somewhat mitigate against this, we examined alcohol consumption self-reported by the same participant in both Waves I and III (i.e., repeated measures) and controlled for consumption reported in both Waves I and III in analyses. In addition, it should be noted that there were only a small number of participants who frequently consumed alcohol at 5 drinks or more in this study. There is no obvious reason to suspect that the findings demonstrated here would not be generalizable to similarly aged populations in the developed nations with broadly similar drinking patterns (e.g., Australia, UK, New Zealand, and Canada) or that the significance of youth drinking will become less pressing in the future. In Australia, for instance, there is evidence to suggest that underage drinking at relatively heavy levels has become increasingly common over the last few decades [12, 34, 35] despite its potential harms for physical and mental development [36]. The findings from this study underscore the importance of preventative approaches to alcohol misuse among adolescents and children.

## Supplementary Material

Details of estimates from multivariate logistic regression models are provided in online supplementary materials.

## Figures and Tables

**Table 1:** Description of participants.

	Diabetes cases	Number of participants
Gender		
Male	73	1,303
Female	99	1,547
Age strata by quartiles (Wave III)		
1st strata	35	739
2nd strata	41	759
3rd strata	42	679
4th strata	54	673
Race		
White	77	2,070
Black	83	624
Other	12	156
Wave I alcohol consumption		
Lifetime abstainer	96	1,368
Current abstainer	9	231
3–7 days/week, <5 drinks/occasion	2	26
3–7 days/week, 5+ drinks/occasion	13	57
2–8 days/month, <5 drinks/occasion	7	175
2–8 days/month, 5+ drinks/occasion	6	220
1–12 days/year, <5 drinks/occasion	33	572
1–12 days/year, 5+ drinks/occasion	6	201
Wave III alcohol consumption		
Lifetime abstainer	54	588
Current abstainer	9	121
3–7 days/week, <5 drinks/occasion	5	112
3–7 days/week, 5+ drinks/occasion	12	157
2–8 days/month, <5 drinks/occasion	28	605
2–8 days/month, 5+ drinks/occasion	21	449
1–12 days/year, <5 drinks/occasion	36	629
1–12 days/year, 5+ drinks/occasion	7	189
Wave III BMI		
<25	49	1451
25–29	43	740
30–34	45	384
≥35	35	275

**Table 2:** Alcohol consumption during adolescence and risk of diabetes, controlling for multiple confounders and using current abstainers as reference group*.

	Odds ratio	95%	CI
Alcohol consumption at Wave I			
Lifetime abstainer^*∧*^	2.97	1.30	6.79
Current abstainer	reference		
3–7 days/week, <5 drinks/occasion	0.98	0.14	6.97
3–7 days/week, 5+ drinks/occasion^*∧*^	12.57	4.10	38.61
2–8 days/month, <5 drinks/occasion	1.39	0.42	4.62
2–8 days/month, 5+ drinks/occasion	0.58	0.16	2.12
1–12 days/year, <5 drinks/occasion^*∧*^	2.46	1.04	5.82
1–12 days/year, 5+ drinks/occasion	1.16	0.35	3.77
Alcohol consumption at Wave III			
Lifetime abstainer	1.56	0.59	4.16
Current abstainer	reference		
3–7 days/week, <5 drinks/occasion	1.03	0.24	4.53
3–7 days/week, 5+ drinks/occasion	1.18	0.35	3.91
2–8 days/month, <5 drinks/occasion	0.88	0.30	2.55
2–8 days/month, 5+ drinks/occasion	0.80	0.26	2.44
1–12 days/year, <5 drinks/occasion	1.04	0.38	2.84
1–12 days/year, 5+ drinks/occasion	0.87	0.24	3.12

*Analysis controlled for the same potential confounders as those listed in Table A1, including gender, age at Wave IV, race, BMI (calculated from measured weight and height) at Wave III, parental self-reported diabetic status, frequency of parental alcohol use (from Wave I), smoking status at Waves I and III, urbanity, and median income of residential location at Wave I.

^*∧*^ *P* value < 0.05.

Confounders are not shown; results were similar to those given in Table A1.

## References

[1] Zimmet P, Alberti KGMM (2001). Global and societal implications of the diabetes epidemic. *Nature*.

[2] Danaei G, Finucane MM, Lu Y (2011). National, regional, and global trends in fasting plasma glucose and diabetes prevalence since 1980: systematic analysis of health examination surveys and epidemiological studies with 370 country-years and 2*·*7 million participants. *The Lancet*.

[3] Lindström J, Louheranta A, Mannelin M (2003). The finnish Diabetes Prevention Study (DPS). *Diabetes Care*.

[4] Eriksson K-F, Lindgarde F (1991). Prevention of Type 2 (non-insulin-dependent) diabetes mellitus by diet and physical exercise The 6-year Malmö feasibility study. *Diabetologia*.

[5] Carlsson S, Hammar N, Grill V, Kaprio J (2003). Alcohol consumption and the incidence of type 2 diabetes: a 20-year follow-up of the finnish twin cohort study. *Diabetes Care*.

[6] Tsumura K, Hayashi T, Suematsu C, Endo G, Fujii S, Okada K (1999). Daily alcohol consumption and the risk of type 2 diabetes in Japanese men: the Osaka Health Survey. *Diabetes Care*.

[7] Baliunas DO, Taylor BJ, Irving H (2009). Alcohol as a risk factor for type 2 diabetes. *Diabetes Care*.

[8] Liang W, Chikritzhs T (2013). Observational research on alcohol use and chronic disease outcome: new approaches to counter biases. *Scientific World Journal*.

[9] Liang W, Zhao Y, Lee AH (2014). A proxy outcome approach for causal effect in observational studies: a simulation study. *Biomed Research International*.

[10] Liang W, Chikritzhs T (2011). Reduction in alcohol consumption and health status. *Addiction*.

[11] Rocchini AP (2002). Childhood obesity and a diabetes epidemic. *The New England Journal of Medicine*.

[12] Liang W, Chikritzhs T (2012). The association between age at first use of alcohol and alcohol consumption levels among adult general drinking population. *Journal of Public Health*.

[13] Harris K (2011). *Design Features of Add Health*.

[14] Harris K, Udry J (2012). *National Longitudinal Study of Adolescent Health (Add Health), 1994–2008 Wave 4, Public Use Biomarkers, Glucose Data Collection Methods*.

[15] Liang W, Chikritzhs T (2013). Alcohol consumption and health status of family members: health impacts without ingestion. *Internal Medicine Journal*.

[16] Chantala K (2006). *Guidelines for Analyzing Add Health Data*.

[17] Arteel GE (2003). Oxidants and antioxidants in alcohol-induced liver disease. *Gastroenterology*.

[18] Liang W, Chikritzhs T, Pascal R, Binns CW (2011). Mortality rate of alcoholic liver disease and risk of hospitalization for alcoholic liver cirrhosis, alcoholic hepatitis and alcoholic liver failure in Australia between 1993 and 2005. *Internal Medicine Journal*.

[19] Marotta F, Barreto R, Wu CC (2005). Experimental acute alcohol pancreatitis-related liver damage and endotoxemia: synbiotics but not metronidazole have a protective effect. *Chinese Journal of Digestive Diseases*.

[20] Sasikala M, Talukdar R, Pavan kumar P (2012). *β*-cell dysfunction in chronic pancreatitis. *Digestive Diseases and Sciences*.

[21] Plum L, Belgardt BF, Brüning JC (2006). Central insulin action in energy and glucose homeostasis. *The Journal of Clinical Investigation*.

[22] Leslie RDG, Pyke DA (1978). Chlorpropamide-alcohol flushing: a dominantly inherited trait associated with diabetes. *British Medical Journal*.

[23] Agrawal A, Freedman ND, Cheng Y-C (2012). Measuring alcohol consumption for genomic meta-analyses of alcohol intake: opportunities and challenges. *The American Journal of Clinical Nutrition*.

[24] Neumark YD, Friedlander Y, Durst R (2004). Alcohol dehydrogenase polymorphisms influence alcohol-elimination rates in a male jewish population. *Alcoholism*.

[25] Thomasson HR, Crabb DW, Edenberg HJ, Li T-K (1993). Alcohol and aldehyde dehydrogenase polymorphisms and alcoholism. *Behavior Genetics*.

[26] Hashimoto Y, Nakayama T, Futamura A, Omura M, Nakarai H, Nakahara K (2002). Relationship between genetic polymorphisms of alcohol-metabolizing enzymes and changes in risk factors for coronary, heart disease associated with alcohol consumption. *Clinical Chemistry*.

[27] Ishikawa H, Yamamoto H, Tian Y, Kawano M, Yamauchi T, Yokoyama K (2003). Effects of ALDH2 gene polymorphisms and alcohol-drinking behavior on micronuclei frequency in non-smokers. *Mutation Research*.

[28] Beulens JWJ, Rimm EB, Hendriks HFJ (2007). Alcohol consumption and type 2 diabetes. *Diabetes*.

[29] Suzuki Y, Taniyama M, Muramatsu T, Ohta S, Atsumi Y, Matsuoka K (2003). Influence of alcohol intake and aldehyde dehydrogenase 2 phenotype on peripheral neuropathy of diabetes. *Diabetes Care*.

[30] Suzuki Y, Muramatsu T, Taniyama M (1996). Mitochondrial aldehyde dehydrogenase in diabetes associated with mitochondrial tRNA (Leu(UUR)) mutation at position 3243. *Diabetes Care*.

[31] Hines LM, Stampfer MJ, Ma J (2001). Genetic variation in alcohol dehydrogenase and the beneficial effect of moderate alcohol consumption on myocardial infarction. *The New England Journal of Medicine*.

[32] Ng Fat L, Shelton N (2012). Associations between self-reported illness and non-drinking in young adults. *Addiction*.

[33] Liang W, Chikritzhs T (2012). Alcohol consumption and health status of family members: health impacts without ingestion. *Internal Medicine Journal*.

[34] Doran CM, Shakeshaft AP, Hall W, Petrie D (2009). Alcohol industry and government revenue derived from underage drinking by Australian adolescents 2005. *Addictive Behaviors*.

[35] Wilks J (1987). Drinking among teenagers in Australia: research findings, problems and prospects. *Australian Drug and Alcohol Review*.

[36] Witt ED (2010). Research on alcohol and adolescent brain development: opportunities and future directions. *Alcohol*.

