# Supplementary material for: Alcohol Consumption during Adolescence and Risk of Diabetes in Young Adulthood
Source: Biomed Res Int. 2014 Mar 17;2014:795741. doi: 10.1155/2014/795741 (PMC3976876; doi:10.1155/2014/795741)
Supplement: Supplementary file 1 — Details of estimates from multivariate logistic regression models are provided in online supplementary materials. [file 795741.f1.pdf]

Supplementary table (online material)

Table A1: Alcohol consumption during adolescence and risk of diabetes, controlling for multiple confounders and using lifetime abstainers as reference group

	Odds Ratio	95%	CI
Wave I alcohol consumption			
lifetime abstainer	reference		
current abstainer^	0.34	0.15	0.77
3-7days/week, < 5drinks/occasion	0.33	0.05	2.12
3-7days/week, 5+ drinks/ occasion^	4.24	1.66	10.83
2-8 days/month, < 5drinks/occasion	0.47	0.18	1.24
2-8 days/month, 5+ drinks/ occasion^	0.20	0.06	0.61
1-12 days/year, < 5drinks/occasion	0.83	0.49	1.40
1-12 days/year, 5+ drinks/ occasion	0.39	0.14	1.05
Wave III alcohol consumption			
lifetime abstainer	reference		
current abstainer	0.64	0.24	1.70
3-7days/week, < 5drinks/occasion	0.66	0.20	2.21
3-7days/week, 5+ drinks/ occasion	0.75	0.31	1.80
2-8 days/month, < 5drinks/occasion	0.56	0.28	1.13
2-8 days/month, 5+ drinks/ occasion	0.51	0.25	1.07
1-12 days/year, < 5drinks/occasion	0.67	0.38	1.18
1-12 days/year, 5+ drinks/ occasion	0.56	0.21	1.47
Gender			
male	reference		
female	1.02	0.67	1.56
Age strata at wave IV separated by quartiles			
1st strata (youngest)	reference		
2nd strata	1.21	0.68	2.16
3rd strata	1.41	0.79	2.53
4th strata	1.75	0.96	3.17
Race			
white	reference		
Black^	3.29	1.98	5.48
Other	1.42	0.67	3.04
Ever smoke regularly: 1+ cigarettes /day (Wave I)			
no	1.40	0.85	2.29
yes	1.61	0.83	3.10
never smoked a whole cigarette	reference		
Ever smoke 1+ cigarettes /day for 30+ days (Wave III)			
no	1.04	0.58	1.89
yes	1.11	0.65	1.90
never smoked a whole cigarette	reference		

BMI (Wave III)			
< 25	reference		
25-29^	1.88	1.12	3.16
30-34^	3.12	1.82	5.33
35+^	3.78	2.19	6.52
Biological mother had diabetes			
No	reference		
Yes	1.27	0.52	3.10
Don't know	1.45	0.52	4.03
Biological father had diabetes			
No	reference		
Yes	1.88	0.96	3.68
Don't know	1.39	0.73	2.66
Father’s alcohol consumption in the past 12 months (Wave I)			
Never^	2.29	1.18	4.45
<= 1/month	reference		
2-3 days/month	1.51	0.62	3.68
1-2 days/week	1.70	0.81	3.57
3-7 days/week	2.10	0.91	4.88
not living with father	1.77	0.91	3.44
Mother’s alcohol consumption in the past 12 months (Wave I)			
never	0.86	0.54	1.38
<= 1/month	reference		
2-3 days/month	0.34	0.14	0.85
1-2 days/week	0.69	0.30	1.60
3-7 days/week	1.15	0.34	3.88
Not living with mother	0.79	0.23	2.77
Urbanity of residential area (Wave I)			
Urban	reference		
not urban	1.39	0.90	2.13
Median household income of residential area by quartiles (Wave I)			
1st strata (lowest)	reference		
2nd strata	1.28	0.77	2.14
3rd strata	1.22	0.67	2.22
4th strata	1.14	0.58	2.24

^p value < 0.05

Table A2: Alcohol consumption during adolescence and parental diabetic status, controlling for multiple confounders and using lifetime abstainers as reference group

	Odds Ratio	95% CI	
Wave I alcohol consumption			
lifetime abstainer	reference		
current abstainer	0.84	0.44	1.61
3-7days/week, < 5drinks/occasion	1.45	0.31	6.73
3-7days/week, 5+ drinks/ occasion	0.71	0.18	2.89
2-8 days/month, < 5drinks/occasion	1.56	0.82	2.95
2-8 days/month, 5+ drinks/ occasion	1.11	0.55	2.24
1-12 days/year, < 5drinks/occasion	1.11	0.68	1.81
1-12 days/year, 5+ drinks/ occasion	0.79	0.39	1.62
Wave III alcohol consumption			
lifetime abstainer	reference		
current abstainer^	2.56	1.28	5.09
3-7days/week, < 5drinks/occasion	1.76	0.67	4.62
3-7days/week, 5+ drinks/ occasion	0.99	0.43	2.29
2-8 days/month, < 5drinks/occasion	1.61	0.94	2.75
2-8 days/month, 5+ drinks/ occasion	1.25	0.65	2.41
1-12 days/year, < 5drinks/occasion	1.38	0.85	2.26
1-12 days/year, 5+ drinks/ occasion	0.92	0.43	1.96

*Analysis controlled for the same potential confounders as those listed in Table A1, but excluding parental self-reported diabetic status from the controlled variable list.

^p value < 0.05
